# Hybrid versus cemented implants for total hip replacement: a randomised feasibility study with embedded qualitative research

**DOI:** 10.1186/s12891-026-09667-3

**Published:** 2026-03-03

**Authors:** Tim N. Board, Vikki Wylde, Hiren Divecha, Matthew Gornall, Richard Jackson, Tony Coffey, Martin Eden, Garima Dalal, Amy Davies, Helen Hickey, Helen Spickett, Tracey Taylor, Matthew Wilson, Rachael Powell

**Affiliations:** 1 Wrightington, Wigan and Leigh Teaching Hospitals NHS Foundation Trust, Wigan, UK; 2 Musculoskeletal Research Unit, Bristol Medical School, University of Bristol, Bristol, UK; 3 NIHR Bristol Biomedical Research Centre, University Hospitals Bristol and Weston NHS Foundation Trust and University of Bristol, Bristol, UK; 4 Liverpool Clinical Trials Centre, University of Liverpool, Liverpool, UK; 5 Manchester Centre for Health Economics, The University of Manchester, Manchester, UK; 6 Division of Nursing, Midwifery and Social Work, School of Health Sciences, University of Manchester, Manchester, UK; 7 Royal Devon University Hospital NHS Foundation Trust, Exeter, UK; 8 Manchester Centre for Health Psychology, School of Health Sciences, University of Manchester, Manchester, UK

**Keywords:** Hip replacement, Feasibility study, Hybrid, Cemented, Qualitative

## Abstract

**Background:**

To evaluate the feasibility of a randomised controlled trial (RCT) comparing the clinical and cost-effectiveness of hybrid versus cemented implants for total hip replacement (THR). Two-arm pragmatic randomised feasibility trial with embedded qualitative semi-structured interviews.

**Methods:**

Performed at two NHS orthopaedic hospitals for the randomised feasibility trial; two further sites for qualitative interviews. 40 adults undergoing THR randomised to receive either cemented (cemented cup and stem) or hybrid (uncemented cup and cemented stem) THR. Qualitative study: 27 patients invited to participate in the trial; 16 consultant orthopaedic surgeons; 4 healthcare professionals involved in trial conduct. Primary outcomes: recruitment rate. Secondary outcomes related to feasibility and acceptability assessed using quantitative and qualitative methods.

**Results:**

February- July 2021: 63 patients were screened and 40 (63%) randomised on a 1:1 allocation which was 100% of the adjusted recruitment target. Four participants were withdrawn due to surgery delays and five of 36 participants did not receive their allocated treatment. No adverse events of special interest and no serious adverse device effects were reported. Data completeness and follow-up were high. An indicative cost-utility analysis was performed, demonstrating feasibility for an RCT. Qualitative findings suggested that trial participation was acceptable to patients if they believed that their well-being would not be compromised. Surgeons’ recommendations regarding trial participation seemed influential. Surgeons seemed willing to recruit patients to the trial if they were in equipoise and perceived no detriment to patients’ outcomes following random allocation.

**Conclusions:**

This study has demonstrated that an RCT to evaluate the clinical and cost-effectiveness of hybrid vs cemented implants for THR is feasible and appears acceptable to patients where their surgeons support trial participation. It is important for surgeons to believe trial participation will not jeopardize patient outcomes to enable surgeons to support the trial.

**Trial registration:**

UK Clinical Study Registry ISRCTN11097021 date of registration 19/2/2021.

**Supplementary Information:**

The online version contains supplementary material available at 10.1186/s12891-026-09667-3.

## Background

Total hip replacement (THR) is one of the most common elective surgical procedures, with over 100,000 operations performed annually in the NHS [1]. The primary aim of THR is to provide relief from chronic pain and reduce functional limitations, most commonly caused by osteoarthritis. Although THR offers generally good outcomes and is deemed a highly cost-effective procedure [2], not all patients achieve their desired functional outcome [3]. Studies have shown that 10–30% of patients may not improve following THR [3, 4]. In the NHS, a number of fixation methods are used in THR surgery; 19% of THRs are cemented (cemented cup and stem), 42% are hybrid (uncemented cup and cemented stem) and 35% are uncemented (uncemented cup and stem) [1]. There is little existing evidence investigating the influence of implant type on functional outcome and there have been recommendations for high quality randomised controlled trials (RCTs) to be performed to evaluate the clinical and cost-effectiveness of different THR fixation methods [5, 6]. However, there are key uncertainties that need to be addressed prior to conducting an RCT.

The aim of the HipHOP study (Hip arthroplasty with Hybrid Or cemented implants: Patient reported outcomes) was to evaluate the feasibility and acceptability of an RCT evaluating the clinical and cost-effectiveness of hybrid vs cemented THR. Our comparison was limited to hybrid vs cemented because the Department of Health’s ‘Getting it Right First Time’ report [7] encourages NHS trusts to move away from fully uncemented THRs due to the lower survivorship and higher treatment costs, and our scoping work with surgeons suggested an unwillingness to use fully uncemented THA in older patients. The objectives of the study were to: assess recruitment, follow-up and withdrawal rates; collect outcomes data to determine the sample size for an RCT; assess intra- and post-operative safety; identify incidence of treatment cross-over during surgery; and determine the feasibility of conducting a within-trial cost-utility analysis. An embedded qualitative study was conducted to understand the experiences of patients, surgeons and research staff, investigating reasons for participation (or declining) and experiences of participating in, or running, the trial.

## Methods

### Study design

HipHOP (Hip arthroplasty with Hybrid Or cemented implants: Patient-reported outcomes).

(Clinical trial registration ISRCTN11097021, registered 19/02/2021) was a multi-centre single-blind randomised feasibility study with embedded qualitative research. Reporting follows guidance from the CONSORT extension for randomised feasibility trials [8] and COREQ guidance [9] for qualitative research; checklists are provided in Additional File 1.

### Patient and public involvement

The study was designed and conducted in collaboration with the Wrightington Wigan and Leigh Patient Research Advisory Group (PRAG). The PRAG were consulted at the grant application stage and advised on the relevance of the topic to patients and the proposed design, including the outcome measures and study processes. Patient-facing documents were developed with the PRAG to ensure that the language and terminology used was suitable, consistent and understandable. Strategies and materials for public dissemination were developed in collaboration with the PRAG.

### Feasibility trial methods

#### Participants

Patients on the waiting list for THR surgery at two NHS orthopaedic hospitals in Wrightington and Exeter were recruited. Eligibility criteria included adults listed for a primary THR because of osteoarthritis; able to communicate in spoken English; and able and willing to give informed consent. Exclusion criteria included previous hip surgery and requirement for complex THR surgery, specifically augmentation of the acetabulum and/or shortening/de-rotational osteotomy of the femur at the time of surgery. Patients were provided with study information and all participants provided informed written consent.

#### Randomisation

Participants were randomised to receive either a fully cemented hip or a hybrid hip implant on a 1:1 basis, stratified by hospital. Randomisation lists were generated using Stata (version 15) by the Liverpool Clinical Trials Centre using randomly permuted blocks with separate lists created for each stratification factor. It was not possible to blind the surgeon or clinical care team. Participants and research staff collecting outcome data were blinded to treatment allocation.

#### Interventions

The only stipulation was fixation method of implants. There were no limitations as to the manufacturer of prostheses used, the head/socket material or diameter. The surgical approach, anaesthetic type, post-operative rehabilitation and other concomitant factors followed normal care for the participating hospital.

#### Assessment

Patient-reported outcomes were assessed pre-operatively (prior to randomisation) and then at 3 months and 6 months post-operative. Outcome measures were the Oxford Hip Score [10], Forgotten Joint Score [11], Self-Administered Patient Satisfaction Scale [12] (administered post-operatively only), EQ-5D-5L [13]. A bespoke Health Resource Use Survey was used to measure use of NHS resources. The original planned follow-up at 6-months post-operative was modified to a range of 3 to 6 month to accommodate the impact of COVID-19 on the capacities at the participating hospitals, along with the effect the pandemic delays had on the study timelines.

#### Outcomes

The primary outcome was recruitment rate. Recruitment of 80% of the target was prespecified as demonstrating the feasibility of progressing to an RCT. Secondary endpoint were incidence of cross-over during surgery (use of another type of implant to that allocated at random), safety data, follow-up rates and data completeness. The feasibility of conducting a within-trial cost-utility analysis (CUA) was assessed using a resource use survey and the EQ-5D-5L questionnaire which was used to generate health-related quality of life (HRQoL) scores using the NICE-recommend tariff [14]. Reasons for declining participation were recorded.

#### Sample size

The planned sample size was 60; this was considered sufficient to estimate our key parameter of recruitment rates with acceptable precision [15, 16]. Following the initial UK COVID-19 lockdown and recommencement of surgery at only two of the three planned hospitals, this target was revised to 40 patients.

#### Statistical analysis

The primary outcome is presented as the recruitment ratio of successful recruitment to eligible patients. Revision rate, infection rate, incidences of treatment cross-over during surgery and rates of missing data were summarised using counts and associated percentages. Withdrawal rates were analysed by counts and percentages or median and interquartile range (IQR). Intervention and resources use cost was calculated for each participant and an indicative CUA was conducted (see Additional File 1). Analysis was performed using SAS (Version 9.4) for statistical analysis and Stata (v14) for economic analysis.

### Qualitative methods

A full description of the qualitative methods and analysis is provided in Additional File 1.

#### Participants

Patients were eligible for inclusion if they had been approached to take part in the HipHOP RCT. Participants were purposively sampled to ensure the sample included: patients who accepted (‘acceptors’) and declined (‘decliners’) participation in the RCT; patients from both RCT sites; patients < and ≥ 50 years old; female and male patients. We aimed to recruit 20–30 patients. Surgeon participants were consultant orthopaedic surgeons recruited from the two RCT sites and two additional sites (in Northern England and Eastern England) which were not involved in the RCT. Purposive sampling ensured the sample included surgeons from both RCT sites who did and did not consent to their patients to the RCT (‘randomising’ and ‘non-randomising’ surgeons), and surgeons from both additional sites (‘interview-only’ surgeons). We aimed to recruit 20–30 surgeons. Health Care Professional (HCP) participants were NHS staff involved in recruiting participants and/or collection of data at both RCT sites; we aimed to invite all interested staff.

#### Data collection

All interviews were conducted by telephone and audio-recorded. Interview schedules were informed by relevant literature and the Theoretical Framework of Acceptability [17], and were reviewed by the PRAG and clinical research team members. Patient ‘Acceptor’ interviews were usually conducted 2–4 weeks post-surgery; ‘decliner’ interviews could be conducted pre-surgery or > 2 weeks post-surgery. Topics covered included experiences of being approached for the study, understanding of the study and reasons for deciding to participate (or not). Surgeon interviews explored beliefs about the treatment options; perceptions and experiences of the trial and randomisation; thoughts about implementation of future guidance resulting from a trial; and reasons for participating (or not) in the HipHOP RCT (trial sites only). HCP interviews explored experiences of recruiting patients; thoughts about patient perceptions of the trial; and experiences of data collection.

#### Analysis

Audio-recordings were transcribed verbatim and identifying details removed. An inductive, data-driven thematic analysis was conducted, structured using the Framework approach [18]. Initially, each group’s data were examined separately. Similarities were found across the datasets, so data from all groups were drawn together, enabling a multi-perspective analysis. Reasons recorded by staff for patients declining trial participation were considered alongside qualitative findings.

## Results

### RCT recruitment and randomisation

**Fig. 1:**
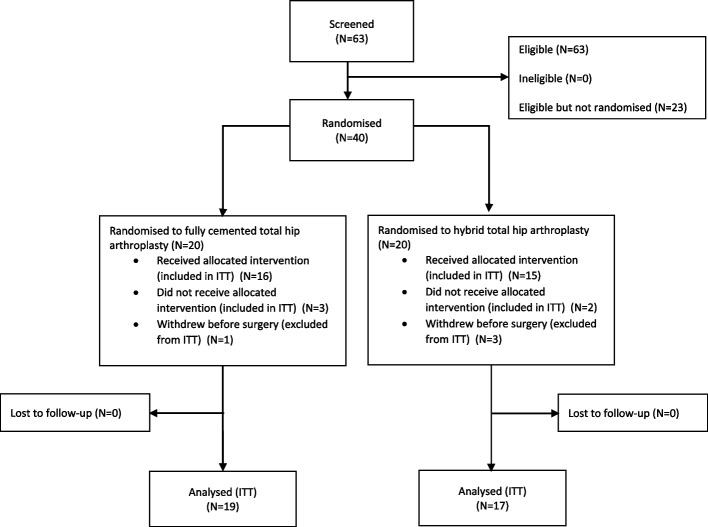
CONSORT flow diagram

An overview of participant flow is provided in Fig. 1. Of the 63 patients screened between February and July 2021, 43 (68%) consented and 40 (63%) were randomised which was 100% of the adjusted recruitment target. Four participants did not receive their surgery within the allocated study window due to the COVID-19 pandemic. Participant baseline characteristics are displayed in Table 1.

**Table 1:** Feasibility study participant characteristics

**Characteristic**		**Fully Cemented** **(*N* = 20)**	**Hybrid** **(*N* = 20)**
Age (years), median (IQR)		64.0 (59.5;72.0)	68.0 (55.0;76.5)
Gender, n (%)	Male	8 (40%)	8 (40%)
	Female	12 (60%)	12 (60%)

### Treatment cross-over

Five participants did not receive their allocated treatment. Two participants allocated to receive hybrid implants received fully cemented due to poor bone quality. Two participants allocated to receive fully cemented implants received hybrid, one due to better-than-expected bone quality and one due to poor bone quality. It should be noted that bone quality was purely the surgeon’s experience-based perception rather than quantitative measurement made intraoperatively. One participant was not suitable for either trial implant due to deterioration in bone stock whilst on the waiting list.

### Safety data

No adverse events of special interest and no serious adverse device effects were reported.

### Follow-up rates and data completeness

Outcome measures were completed by all participants at all time points.

### Feasibility of conducting a cost-utility analysis

An indicative CUA was performed, demonstrating feasibility of conducting an economic evaluation alongside the definitive RCT (see Additional File 1).

### Qualitative findings

Twenty-seven patients (22 ‘acceptors’, 5 ‘decliners’), 16 surgeons and 4 HCPs were interviewed (Table 2). Further sample information is provided in Additional File 1.

**Table 2:** Interview participant characteristics

Characteristic		Participants/participant characteristics
Patient sample (*n* = 27)		
Participation in HipHOP trial		
‘Acceptor’		22
‘Decliner’		5
Surgery site		
Site A		21
Site B		6
Previously received hip replacement		6
Time between surgery and interview (days post-surgery)		Median: 23 (range: 18 to 56)
Interview duration (minutes)		Median: 32 (range: 20 to72)
Gender		
Female		15
Male		12
Age (years)		Median = 63 (range: 40 s to 80 s)
Ethnic group		
White British		27
Surgeon sample (*n* = 16)		
Participation in HipHOP trial*		
Trial recruitment site	10	
(‘Randomising’)	(4)	
(‘Non-randomising’)	(6)	
Interview-only site	6	
Number of hip replacement operations surgeons reported typically performing each month†	Median = 15.85 (range: ≤ 10 to ≥ 30)	
Number of years at consultant grade	Median = 14 (range: ≤ 5 to ≥ 25)	
Number of years practicing hip replacement surgery	Median = 16.5 (range: ≤ 10 to ≥ 30)	
Interview duration (minutes)	Median: 29 (range: 18 to 49)	
HCP sample (*n* = 4)		
Years experience in current role	Median = 3‡	
Years experience recruiting patients for research studies	Median = 3‡	
Participant estimation of number of patients had worked with on HipHOP feasibility trial	Median = 14‡	
Interview duration (minutes)	Median: 41 (range: 30 to 57)	

^*^ ‘Randomising’: surgeons who agreed to recruit their patients to the feasibility trial and for them to be randomly allocated to implant group; ‘non-randomising’: surgeons whose patients were not recruited to the trial; Interview-only site: surgeons were based at sites which did not recruit patients into the feasibility trial

^†^Where surgeons reported numbers as a range, mean average between the two figures was used

^‡^Ranges not presented to avoid identification risk in small population

Reasons recorded for patients declining trial participation are presented in Table 3. Most individuals did not give a clear reason for declining participation.

**Table 3:** Reasons for patients declining participation in the trial arm of HipHOP (across both sites)

Category	Occurrences	Details
No clear reason given	14	Included: no reason given (9); stating not interested/not wishing to take part (4); declined after discussion with family (1)
Feels too old	1	
Too busy (work/caring commitments)	2	
Not wishing to be allocated to treatment at random	2	Not comfortable with randomisation (1); had good results with one implant type previously (1)

Four themes are presented. Findings related to the themes ‘Acceptability of data collection’ and ‘Desire to help’ are of general relevance, so are briefly summarised here with full content in Additional File 1. We found discussions around randomisation and equipoise to be important across groups; these discussions were developed into inter-related themes presented in full below: ‘Patient perspectives on treatment allocation’ and ‘Equipoise: Surgeon and HCP beliefs’. A further area of importance was considerations for implementing future trial findings in practice; findings related to implementation are reported elsewhere [19].

#### Acceptability of data collection

Most ‘acceptor’ participants seemed to have few concerns about completing trial processes, and questionnaire completion seemed broadly acceptable. There was some indication in both HCP and patient interviews that having no extra hospital visits was appreciated, but some participants recognised that other commitments might impact capacity to complete questionnaires. Two individuals reported to staff that they declined trial participation because of being too busy (Table 3). ‘Decliner’ interview participants generally indicated that they would have been happy to complete questionnaires; other aspects of the study such as treatment allocation seemed more off-putting.

#### Desire to help

The desire to help improve future treatment for patients seemed important and influential in many participants’ decision to participate in the trial. Some individuals declined trial participation despite appearing pro-research. It seemed that an enthusiasm for research participation could lead to conflict for ‘decliners’ who wished to support research but held concerns about participating in this specific RCT. A sense of obligation around participation was apparent for some participants. Individuals seemed to value receiving surgery and appeared glad to be able to reciprocate and ‘give back’ to the NHS and their care teams.

#### Patient perspectives on treatment allocation (see Table 4 for supporting quotations)

**Table 4:** Quotations supporting reported qualitative findings

Theme & summary of concept	Supporting quotations
*Theme—Patient perspectives on treatment allocation*	
Comfort with allocation process	
• Perceiving little difference between treatments such that not putting selves at risk	*[the surgeon] did reassure me that, you know, that they don’t know what’s best, and so to be randomised wasn’t going to put me at a disadvantage. And that for a lot of people it, you know, they don’t believe at the moment it makes a huge difference as to which one you get, and that that’s what they’re trying to research to see if there is a difference. So you know, [the surgeon] could have easily done a cemented one or a non-cemented one without the trial, and so it wasn’t a burden for me to carry into be part of the trial really.* (Patient LS, acceptor)
• HCP perception patients comfortable with either implant	*everyone seemed to be happy, you know, these two things were in circulation and nobody knew which was better […] I guess there must have been a feeling of they’re safe to put in and they’re fine.* (HCP W)
• Understanding both implants to be approved devices	*I felt that nobody would be putting a hip into me under the NHS, if they didn’t think either hip was a good option. So I realised it wasn’t like, let’s try [me] with something that’s never been tried before, on a person. So I was quite happy to go with either one* (Patient ZR, acceptor)
• Random allocation could be off-putting	*Well, randomisation was probably the main one [reason for not wishing to take part]* (HCP F)
• Desire to receive best treatment for individual characteristics	*there must be different situations with age, size of people, you know, just things like that, I think that should be taken into consideration. And I think it should be clinical, rather than just sort of like a lucky dip if that’s the way to look at it.* (Patient VR, decliner)
• Desire new implant to be same as previous implant – keep with what works	*I didn’t really see why I would have something different* (Patient VR, decliner)
• Reluctance to recruit patients with previous implant to trial	*a patient who comes with a fixed mind of what they believe they need – or a patient who has had a cemented or a hybrid hip on the other side and it’s been very successful – I would not ask them to be randomised into a trial. […] If they have a poorer outcome on the second side, they will blame the fact it’s a different implant.* (Surgeon A, non-randomising)
Understanding of randomisation	
• Good understanding of randomisation	*It means that the decision-making process is out of the surgeon’s hand, and the programme says yes [the patient] has cement, or no [the patient] doesn’t have cement. And it’s not based on any clinical information, apart from the fact that it’s being decided that either would be okay for me.* (Patient LS, acceptor)
• Perception randomisation consistent with surgeon deciding treatment	*[the surgeon] did two different styles and would I be on a survey, [the surgeon] did what he thought was the right one, and would I mind being part of it* (Patient VO, acceptor)
• Perception computer deciding treatment based on individual data	*obviously someone’s got to put something into the computer to give an answer back, I thought, “well, as long as all that’s put in correctly, and the computer then picks out the correct way to go, then obviously if the surgeon had any doubts he would obviously override the computer” […] I mean computers can only give out what they’re put in.* (Patient UG, acceptor)
• HCPs finding language emphasising chance nature of allocation either useful or to be avoided	*I think when you say the word ‘randomisation’, they’re a bit like oh, okay, whereas if you sort of use the terminology, ‘flipping a coin’ or ‘rolling a dice’, […] the patients seemed to get it more* (HCP Q) *some people say like the toss of a coin, but I don’t normally use that one because it seems like a bit of a gamble.* (HCP C)
Patients’ trust in surgeons	
• Trust in surgeon an important influencing factor	*I know [name of surgeon] is very good, and you just put your trust into, you know, what they think.* (Patient VO, acceptor)
• Surgeon recommending trail indicative that trial participation is good option for them	*I think that the thing that just gave me the go ahead was the fact that, you know, they were going to assess that either would be equally appropriate for me. And so to be randomised at that point wasn’t really a massive risk to take* (Patient LS, acceptor)
• Reassured by belief that surgeon would not use allocated implant if judged alternative to be better option	*Initially, I thought, well, you know, it’s potluck. But I was assured the surgeon would have the final say.* (Patient KC, acceptor) *if the surgeon had any doubts [the surgeon] would obviously override the computer, I thought. Because obviously if the surgeon thinks, no, that’s not right in this instance, I would assume – hope – that they would do the right thing there* (Patient UC, acceptor)
• HCPs reinforced understanding that surgeons would not risk patient well-being for trial purpose	*So I normally say that the doctors have identified that you’d be eligible for either, and – but if for any reason on the day of surgery they decide that would be better for you than the other, then they would withdraw you from the study and you’d have the one that would be safest for you.* (HCP C)
• Surgeon equipoise could facilitate conversations about recruitment	*a couple wanted to speak to me, they would ask, “Well which one would you have,” or “Which do you think is the best for me?” And the advantage of having genuine equipoise was because I could actually say, “I really don’t know and I don’t think it matters”* (Surgeon O, randomising)
• Surgeon preconception about best treatment could make recruitment conversations challenging	*Because patients are good at picking up your feelings about one thing or the other. And this is the point that I would find, I think, quite difficult to say to a patient that, you know, I really don’t think one is better than the other. So, but I think if patients are counselled by people who have true equipoise about the treatment, then I think they will feel – they will share that equipoise* (Surgeon T, non-randomising)
*Theme—Equipose: Surgeon and HCP beliefs*	
Equipoise and randomisation of patients	
• Surgeon perception of equipoise linked with willingness to be involved in trial	*I think one of the reasons I was very happy to become a recruiting surgeon was because I already had quite a lot of equipoise.* (Surgeon O, randomising) *I’d be very happy to join in with that, and it’s – would answer a question that I’ve always wanted to ask, so I’d be very comfortable in doing either in any patients that were randomised, and you know, being confident that I wasn’t doing them a disservice.* (Surgeon V, interview-only site) *on reflection I didn’t feel that I had sufficient equipoise to do a randomised control trial of this choice, particularly in patients under the age of fifty. I just – I just didn’t think I’d be honest to myself if I took part* (Surgeon T, non-randomising)
• Examples of surgeons preferring particular implant for patients with specific condition	*patients with inflammatory arthropathy. I don’t think they should have a hybrid because the inflammatory arthropathies do better with cemented implants* (Surgeon N, non-randomising) *So [patients with] rheumatoid arthritis, […] potentially I might be more inclined to use a cemented component for them. […] for most of the patients I operate on, provided their anatomy is good enough to have the option of both, it could be either.* (Surgeon M, interview-only site)
• Variation in age range where surgeons perceived equipoise	*I think there’s not equipoise in the 90-year-olds. I think the community would say it would be foolish to do an uncemented socket in a 90-year-old.* (Surgeon J, interview-only site) *one of the major increased risks if you like of uncemented, is fracture, intraoperative fracture and that’s with increasing age and poorer quality of bones. So, at some point you have to have a cut-off as to when you would not do uncemented sockets. […] I would guestimate with your average person over the age of seventy say, you probably shouldn’t* (Surgeon G, non-randomising)
• Surgeons less comfortable to randomise in age ranges where lacked equipoise	*the only concern I had was for patients of the extreme of age. So patients under the age of thirty, I would be concerned about putting a cemented component into those patients. And, likewise, the opposite for patients above, eighty to eight-five I’ll have concerns. So I was more reluctant to recruit those to the trial.* (Surgeon Z, randomising)
• Example of surgeon seeming in conflict – but would still consider trial participation	*I think I would try to overcome my sort of personal anxiety […] because I know in myself, I’m a good surgeon and I get good results with both. […] I think the research elements give you some sort of protection. Say, for example, the [specific implant] group were found to do less well […] Then I guess, in terms of the trial they understood the risks of it and then I guess, it's not great, but I guess I'll be happy to recruit to that trial I think.* (Surgeon C, randomising)
Surgeon competence: Ethics and equipoise	
• Perceived competency with implant types impacted by training and experience	*I think the barriers will be where consultants are in their clinical practice and in their training. […] There are some surgeons – and probably the older type, that are predominantly cemented […] if you talk to some of the trainees now, because there’s been a growth in cementless implants, a lot of them very rarely cement an implant in.* (Surgeon A, non-randomising)
• Perceived competency with an implant as factor in declining participation in feasibility trial	*I wouldn’t want my patients to be randomised […] In my hands the best results are with cemented, so if they stay on my waiting list it will be a cemented hip replacement.* (Surgeon N, non-randomising)
• Concern of negative impact on patients and trial findings where surgeon lacks skill in using an implant type	*if there’s no selection of the surgeon group who are asked to participate, it’s not ethical. […] if I was involved in this study, and I was asked to randomise my patients […] that’s not ethical in my view. Because I’ve been asked to do something I’ve not been trained to do. And I’m also introducing distortion in the study.* (Surgeon A, non-randomising)
• Possibility that patients could receive implant from another surgeon where competency an issue	*I would only be happy if patients who wanted to be recruited to that trial had their care transferred to a surgeon who was comfortable doing both cemented sockets and uncemented sockets. And I wouldn’t have a problem with that as long as it was like, the whole process is transparent.* (Surgeon A, non-randomising)
• Challenges with transferring patients to other surgeons: trust in surgeon–patient relationship and resource issues	*forty to fifty percent of the patients that I operate on are my old patients […] So I suspect they’ll be somewhat reluctant to leave me and go to somebody else.* (Surgeon N, non-randomising) *I’m not sure how that would go down with patients […] when you have built up a rapport with them in the clinic, I mean to then be operated on by somebody else I think they would have to be seen in clinic by somebody else as well, which is not the most efficient system to do it on.* (Surgeon G, non-randomising)

##### Comfort with allocation process

Many ‘acceptor’ participants seemed comfortable with randomisation. It seemed that believing there to be little difference between the treatments was important, such that individuals did not feel they were putting themselves at risk. HCP participants also perceived that many patients felt comfortable to receive either implant. It seemed that understanding both implant types to be approved, routinely used, and endorsed by surgeons and NHS hospitals contributed to individuals believing either option to be good for them. This seemed to diminish perceptions of risk in participating and help individuals to feel they were being cared for rather than experimented on. However, it appeared that randomisation could be off-putting for some patients. Perceiving there to be a difference between the implants – and the desire to avoid risk – seemed a factor in declining participation. For example, some ‘decliners’ appeared to focus on ensuring that the implant they received was the best treatment for their individual characteristics. Some decliners were individuals who had previously received a successful hip implant and wanted the same implant this time. For one surgeon, concern about how a patient’s previous experience might influence perceptions about randomisation seemed to lead to reluctance to recruit patients who had previously had a hip replacement.

##### Understanding of randomisation

Some patient participants seemed to have a good understanding of randomisation, but others appeared to think randomisation was consistent with a decision being made by the surgeon as to which type of implant would be best for them as an individual. For some, the concept of the allocation being made by computer seemed to contribute to misunderstandings. Some seemed to believe that the computer was considering their individual data before indicating allocation, removing the decision from the surgeon, but still deciding what would be best for them as an individual. Some HCPs found using language emphasising the chance nature of allocation to be useful; others sought to avoid language associated with gambling.

##### Patients’ trust in surgeons

For many individuals who participated in the trial, trust in their surgeon seemed an important influencing factor. It appeared that some ‘acceptors’ viewed the surgeon recommending the trial as indicative that participation would be a good option for them, and their best interests were still being prioritised. Patient participants also seemed to find reassuring a belief that, should the surgeon think their allocated implant was not the best treatment for them, they would instead receive an implant based on the surgeon’s clinical judgement. It seemed that HCPs involved in recruitment reinforced the understanding that surgeons would not risk patients’ well-being for the trial.

The extent to which the surgeon had equipoise seemed to potentially either facilitate conversations about randomisation or make it challenging. Where a surgeon had equipoise, it seemed that they would typically feel comfortable with having conversations encouraging participation. It seemed that conversations about randomisation could be more difficult where surgeons held a preconception about which treatment would be best. Thus, a trusting relationship between patient and surgeon seemed crucial for patients to feel comfortable to participate in the trial, with patients seeking an honest assessment as to whether participating would affect their receiving optimum treatment. For surgeons, it seemed that a genuine perception of equipoise enabled them to be able to answer patients’ questions openly and honestly whilst being supportive of the trial.

#### Equipoise: surgeon and HCP beliefs

##### Equipoise and randomisation of patients

Some surgeons, across all three groups (randomising, non-randomising and interview-only site) indicated a general perception of equipoise across implant types. Perceiving equipoise often seemed to be associated with willingness to be involved in a trial. Even where equipoise in general was perceived, there were suggestions for exclusion criteria to ensure that only patients who were suitable for either implant would be recruited. A number of surgeons considered anatomical reasons for preferring a particular implant, and inflammatory arthropathy, osteoporosis and rheumatoid arthritis were conditions linked with a reluctance to randomise. Some surgeons perceived equipoise for certain age groups but not for others. There tended to be a favouring of uncemented sockets for younger patients and cemented for older individuals, but the range of ages where equipoise was perceived varied. Surgeons seemed less comfortable about patients being randomised in age ranges where they lacked equipoise.

On occasion, surgeons seemed to experience conflict between feeling they should participate in the research, and not feeling themselves to entirely be in equipoise. One ‘randomising’ surgeon seemed to experience unease when performing a type of surgery which they would not normally choose for a specific individual. Despite this it seemed that this surgeon would still consider participating in a trial, albeit with some reluctance, appearing to feel that the structure of research, and the process of informed consent, would ensure that patients understood the risks of participation. It would seem that there might be tension between patient perceptions that participation would be a good option because the surgeon is supporting the trial – and surgeon perceptions that if individuals participate, they understand there might be associated risks.

##### Surgeon competence: ethics and equipoise

Surgeons seemed to consider equipoise from two perspectives: which implant might work best in general, and which implant might be most effective for a specific surgeon. There was a belief that a surgeon’s results would be affected by their competency in using an implant type, with competency impacted by training and experience. Some surgeons considered competency when discussing their own experience and willingness to participate in a trial. Considering oneself to lack competency in using one implant seemed to be a key factor in declining participation in HipHOP for some surgeons.

Concerns were raised about how the trial might be conducted to ensure all participating surgeons would be competent with both implant types. From an ethical perspective, there was concern that some patients might have poor results due to a surgeon being insufficiently experienced in a particular technique. Concerns were also raised that including surgeons who lacked experience in a technique could lead to uncertainty in trial results, making it difficult to ascertain whether sub-optimal results were due to implant type or surgeons’ lack of skill.

Where surgeons felt that the trial was important but that they did not feel sufficiently competent in using one implant, the possibility that their patients could receive surgery from another surgeon was considered. There was some evidence of willingness for this to occur. However, surgeon participants felt that patients developed trust in their surgeon, and might be unwilling to transfer to another surgeon. There was also a perception that transferred patients would want to meet their new surgeon to build trust, but this would lead to delays in surgery, which was considered to be unwelcome to patients, as well as requiring additional resource.

## Discussion

This feasibility study with embedded economic evaluation and qualitative work has demonstrated that an RCT comparing fixation methods for THR is feasible and acceptable to patients and surgeons. Qualitative findings indicated trial participation seemed broadly acceptable, so long as patients have appropriate time, space and support to complete study procedures. Some concerns around equipoise and surgical skills were identified by surgeon participants; these concerns would need to be addressed when designing the RCT. Intra- and post-operative data collection was robust and patients were able to complete the required functional outcome questionnaires. Our study demonstrated it was possible to collect all requisite data to facilitate a CUA.

The majority of orthopaedic studies are ranked level 3 or below on the level of evidence scale [20]. Literature reviews have only identified a small number of RCTs comparing the outcomes of the different types of THR. A meta-analysis found there were only four RCTs looking at patient outcomes in terms of implant fixation [6], with only one of these studies evaluating the functional outcome of UK patients. A consistent criticism of these RCTs is that they have inconsistent results, poor reporting and missing data thus reducing the power and validity of the conclusions that are presented. A recent systematic review highlights this lack of published evidence and highlighted the need for new trials with rigorous reporting on core, adequately powered outcomes and long-term follow-up to evaluate the effectiveness of different types of implants [5]. There is currently a programme of work ongoing to evaluate the clinical and cost-effectiveness of different fixation methods in younger patients undergoing THR (https://fundingawards.nihr.ac.uk/award/NIHR203671).

Our qualitative findings are broadly in line with previous research. Two systematic reviews identified that individuals may carefully consider trial participation, being motivated to participate through a wish to help others, or to progress medical science, but being reluctant to risk allocation to treatment which could be detrimental to their well-being [21, 22]. These reviews similarly identified trust in their care (and research) teams to be important for individuals to feel comfortable about participation in trials. The present study identified some confusion for patient participants around what randomisation meant. For example, where computer involvement was mentioned, individuals could think that the computer was allocating them to the treatment that would best fit them. This issue has been identified in other surgical trial contexts [22]. Language emphasising the chance nature of allocation, such as referring to coin-tossing, may be useful, and such language features in national information guidance – ‘akin to drawing lots, tossing a coin or rolling a die’[23]. However, this language might not be acceptable to all as some patients could feel uncomfortable with such chance-focussed language [24].

The importance of perceiving equipoise for surgeons to be comfortable to randomise patients has been noted elsewhere [25]. Surgeons wishing to keep to procedures which they believe work for them, in which they have personal experience, was reported in breast cancer surgery, along with the potential conflict of valuing trial evidence but feeling unwilling to risk poor outcomes for a surgeon’s own clinical practice [26]. As patients seem highly influenced by the view of surgeons in considering whether or not trial participation is a good option for them, and surgeons seem comfortable in recommending the trial where they are in equipoise, a future trial should work with surgeons to support a shared, evidence-based perception of equipoise, such that it is clear to surgeons that trial inclusion criteria does not jeopardise the likelihood of good outcomes for patients. There should also either be clear criteria to ensure that surgeons have appropriate expertise to use either implant, or consideration should be given to the management of patients whose surgeons are competent in using only one implant type.

There was some cross-over from allocated to received implant group related to patients’ bone quality. Decisions around bone quality, appropriateness of implant, and whether or not to follow trial allocations were clinical choices made by the operating consultant surgeon. In our qualitative findings, concerns related to bone quality (osteoporosis, age) seemed to be associated with a lack of equipoise in surgeons and a reluctance to allocate participants to implant type via randomisation. In a related paper, where we considered issues relevant to guideline implementation within the same sample of surgeon participants, we reported further discussion around the impact of bone quality on clinical decisions [19]. Aspects associated with bone quality, such as older age, seemed to influence implant preferences for many surgeon participants, with a general preference for hybrid implants in patients with higher bone density (such as younger age groups) and fully cemented in those with lower bone density (e.g. older patient groups). However, there appeared to be individual differences in how factors such as age impacted surgeon’s clinical decisions [19]. Prior to conducting a full trial, it would be useful to work with surgeons to further understand their concerns related to bone quality, and to provide suitable training and guidance within the trial to ensure that surgeons can be confident and consistent in following trial treatment allocation.

A key strength of this study is that it addresses the priorities of patients. The James Lind Alliance has produced a list of the top 10 priorities for research in hip and knee replacement including the question “What (health service) pre-operative, intra-operative, and post-operative factors can be modified to influence outcome following hip and knee replacement?”, which aligns with the aims of our feasibility study. Another strength is that the qualitative study successfully recruited participants with a range of backgrounds, including patients and surgeons who declined participation in the trial and participants from multiple sites. However, we are unlikely to have fully captured the perspectives of individuals who did not wish, or who were unable, to take part in research in general as they were unlikely to have consented to an interview.

The results from this feasibility study support the delivery of a larger trial to evaluate efficacy between hip replacement constructs. However, as this feasibility study was conducted in two sites, some caution is required in extrapolating these results to a large confirmatory study requiring many sites. In that scenario either an intermediate study to measure efficacy (e.g. phase II) which can also collect further feasibility data or a study apply alternative causal inference tools to registry datasets may be valid alternatives.

## Conclusions

By undertaking feasibility work to address key uncertainties, this study has generated important data on the likely success of a future RCT and provided insight into approaches to optimise trial design and processes. Ensuring that patients have suitable resources and support to complete study requirements, and addressing concerns identified by surgeon participants, may enhance both recruitment and retention in a future trial. National guidance is currently unable to make clear recommendations on implant types in hip replacement surgery. Our work has shown that an RCT of hybrid vs cemented hip replacements is acceptable to patients and surgeons and is a feasible undertaking. High quality health resource use and quality of life data can be collected in this patient group and used to calculate an economic model of cost effectiveness. A future RCT would inform national practice by providing evidence to guide decision-making around THR surgery and inform service provision.

## Supplementary Information

.Additional file 1. Part 1: CONSORT extension checklist for information to include when reporting a pilot or feasibility study. Part 2: COREQ checklist for interviews and focus groups. Part 3: Feasibility of data collection and indicative cost utility analysis. Part 4: Details of qualitative research methods and analysis. Part 5: Additional qualitative findings details.

## Data Availability

The datasets used and analysed during the current study are available from the corresponding author on reasonable request. However, in line with the ethical approval of the study and to protect the identity of research participants, qualitative data will not be available for sharing due to risk of participant identification.
