# Supplementary material for: Hybrid versus cemented implants for total hip replacement: a randomised feasibility study with embedded qualitative research
Source: BMC Musculoskelet Disord. 2026 Mar 3;27:284. doi: 10.1186/s12891-026-09667-3 (PMC13064185; doi:10.1186/s12891-026-09667-3)
Supplement: Supplementary file 1 — .Additional file 1. Part 1: CONSORT extension checklist for information to include when reporting a pilot or feasibility study. Part 2: COREQ checklist for interviews and focus groups. Part 3: Feasibility of data collection and indicative cost utility analysis. Part 4: Details of qualitative research methods and analysis. Part 5: Additional qualitative findings details. [file 12891_2026_9667_MOESM1_ESM.docx]

**Additional File 1**

Contents

[Part 1: CONSORT extension checklist for information to include when reporting a pilot or feasibility study 2](#_Toc164945473)

[Part 2: COREQ checklist for interviews and focus groups 6](#_Toc164945474)

[Part 3: Feasibility of data collection and indicative cost utility analysis 9](#_Toc164945475)

[Part 4: Details of qualitative research methods and analysis 12](#_Toc164945476)

[Part 5: Additional qualitative findings details 24](#_Toc164945477)

# Part 1: CONSORT extension checklist for information to include when reporting a pilot or feasibility study

| Section/Topic | Item No | Checklist item | Reported on page No |
| --- | --- | --- | --- |
| Title and abstract | | | |
|  | 1a | Identification as a pilot or feasibility randomised trial in the title | Title |
|  | 1b | Structured summary of pilot trial design, methods, results, and conclusions (for specific guidance see CONSORT abstract extension for pilot trials) | Abstract |
| Introduction | | | |
| Background and objectives | 2a | Scientific background and explanation of rationale for future definitive trial, and reasons for randomised pilot trial | Background |
|  | 2b | Specific objectives or research questions for pilot trial | Background |
| Methods | | | |
| Trial design | 3a | Description of pilot trial design (such as parallel, factorial) including allocation ratio | Methods, randomisation section |
|  | 3b | Important changes to methods after pilot trial commencement (such as eligibility criteria), with reasons | N/A |
| Participants | 4a | Eligibility criteria for participants | Methods, participants |
|  | 4b | Settings and locations where the data were collected | Methods, participants |
|  | 4c | How participants were identified and consented | Methods, participants |
| Interventions | 5 | The interventions for each group with sufficient details to allow replication, including how and when they were actually administered | Methods, interventions |
| Outcomes | 6a | Completely defined prespecified assessments or measurements to address each pilot trial objective specified in 2b, including how and when they were assessed | Methods, outcomes |
|  | 6b | Any changes to pilot trial assessments or measurements after the pilot trial commenced, with reasons | Methods, assessment |
|  | 6c | If applicable, prespecified criteria used to judge whether, or how, to proceed with future definitive trial | Methods, outcomes |
| Sample size | 7a | Rationale for numbers in the pilot trial | Methods, sample size |
|  | 7b | When applicable, explanation of any interim analyses and stopping guidelines | N/A |
| Randomisation: |  |  |  |
| Sequence  generation | 8a | Method used to generate the random allocation sequence | Methods, randomisation section |
|  | 8b | Type of randomisation(s); details of any restriction (such as blocking and block size) | Methods, randomisation |
| Allocation  concealment  mechanism | 9 | Mechanism used to implement the random allocation sequence (such as sequentially numbered containers), describing any steps taken to conceal the sequence until interventions were assigned | Methods, randomisation |
| Implementation | 10 | Who generated the random allocation sequence, who enrolled participants, and who assigned participants to interventions | Methods, randomisation |
| Blinding | 11a | If done, who was blinded after assignment to interventions (for example, participants, care providers, those assessing outcomes) and how | Methods, randomisation |
|  | 11b | If relevant, description of the similarity of interventions | Methods, interventions |
| Statistical methods | 12 | Methods used to address each pilot trial objective whether qualitative or quantitative | Methods, analysis sections |
| Results | | | |
| Participant flow (a diagram is strongly recommended) | 13a | For each group, the numbers of participants who were approached and/or assessed for eligibility, randomly assigned, received intended treatment, and were assessed for each objective | Results section and Figure 1 |
|  | 13b | For each group, losses and exclusions after randomisation, together with reasons | Results section and Figure 1 |
| Recruitment | 14a | Dates defining the periods of recruitment and follow-up | Results, recruitment and randomisation |
|  | 14b | Why the pilot trial ended or was stopped | N/A |
| Baseline data | 15 | A table showing baseline demographic and clinical characteristics for each group | Table 2 |
| Numbers analysed | 16 | For each objective, number of participants (denominator) included in each analysis. If relevant, these numbers  should be by randomised group | Results section and figures/tables |
| Outcomes and estimation | 17 | For each objective, results including expressions of uncertainty (such as 95% confidence interval) for any  estimates. If relevant, these results should be by randomised group | Tables |
| Ancillary analyses | 18 | Results of any other analyses performed that could be used to inform the future definitive trial | Results section and tables |
| Harms | 19 | All important harms or unintended effects in each group (for specific guidance see CONSORT for harms) | Results, safety data |
|  | 19a | If relevant, other important unintended consequences | N/A |
| Discussion | | | |
| Limitations | 20 | Pilot trial limitations, addressing sources of potential bias and remaining uncertainty about feasibility | Discussion |
| Generalisability | 21 | Generalisability (applicability) of pilot trial methods and findings to future definitive trial and other studies | Discussion |
| Interpretation | 22 | Interpretation consistent with pilot trial objectives and findings, balancing potential benefits and harms, and  considering other relevant evidence | Discussion |
|  | 22a | Implications for progression from pilot to future definitive trial, including any proposed amendments | Discussion |
| Other information | | |  |
| Registration | 23 | Registration number for pilot trial and name of trial registry | Abstract |
| Protocol | 24 | Where the pilot trial protocol can be accessed, if available | Not available |
| Funding | 25 | Sources of funding and other support (such as supply of drugs), role of funders | Funding section |
|  | 26 | Ethical approval or approval by research review committee, confirmed with reference number | Ethics approval section |

# Part 2: COREQ checklist for interviews and focus groups

| **Number** | **Item** | **Guide question/description** | **Location in paper** |
| --- | --- | --- | --- |
| **Domain 1: Research team and reflexivity**  *Personal characteristics* | | | |
| 1 | Interviewer/facilitator | Which author/s conducted the interview or focus group? | Supplementary material |
| 2 | Credentials | What were the researcher’s credentials? E.g. PhD, MD | Supplementary material |
| 3 | Occupation | What was their occupation at the time of the study? | Supplementary material |
| 4 | Gender | Was the researcher male or female? | Supplementary material |
| 5 | Experience and training | What experience or training did the researcher have? | Supplementary material |
| *Relationship with participants* | | | |
| 6 | Relationship established | Was a relationship established prior to study commencement? | Supplementary material |
| 7 | Participant knowledge of the interviewer | What did the participants know about the researcher? E.g. personal goals, reasons for doing the research? | Supplementary material |
| 8 | Interviewer characteristics | What characteristics were reported about the interviewer? E.g. bias, assumptions, reasons and interests in the research topic | Supplementary material |
| **Domain 2: Study design**  *Theoretical framework* | | | |
| 9 | Methodological orientation and theory | What methodological orientation was stated to underpin the study? E.g. grounded theory, discourse analysis, ethnography, phenomenology, content analysis | Qualitative study methods – Analysis;  Supplementary material |
| 10 | Sampling | How were the participants selected? E.g. purposive, convenience, consecutive, snowball | Qualitative study methods – Participants;  Supplementary material |
| 11 | Method of approach | How were participants approached? E.g. face-to-face, telephone, mail, email | Supplementary material |
| 12 | Sample size | How many participants were in the study? | Results: Qualitative findings |
| 13 | Non-participation | How many people refused to participate or dropped out? Reasons? | Supplementary material |
| *Setting* | | | |
| 14 | Setting of data collection | Where was the data collected? E.g. home, clinic, workplace | Qualitative study methods – Data collection; Supplementary material |
| 15 | Presence of non-participants | Was anyone else present besides the participants and researchers? | Supplementary material |
| 16 | Description of sample | What were the important characteristics of the sample? E.g. demographic data, date | Results: Qualitative findings (Table 2) |
| *Data collection* | | | |
| 17 | Interview guide | Were questions, prompts, guides provided by the authors? Was it pilot tested? | Qualitative study methods: Data collection |
| 18 | Repeat interviews | Were repeat interviews carried out? If yes, how many? | Supplementary material |
| 19 | Audio/visual recording | Did the research use audio or visual recording to collect the data? | Qualitative study methods: Data collection; Supplementary material |
| 20 | Field notes | Were field notes made during and/or after the interview or focus group? | Supplementary material |
| 21 | Duration | What was the duration of the interviews or focus groups? | Results: Qualitative findings (Table 2) |
| 22 | Data saturation | Was data saturation discussed? | Supplementary material |
| 23 | Transcripts returned | Were transcripts returned to participants for comment and/or correction? | Supplementary material |
| **Domain 3: Analysis and findings**  *Data analysis* | | | |
| 24 | Number of data coders | How many data coders coded the data? | Supplementary material |
| 25 | Description of the coding tree | Did authors provide a description of the coding tree? | Supplementary material |
| 26 | Derivation of themes | Were themes identified in advance or retrieved from the data? | Supplementary material |
| 27 | Software | What software, if applicable, was used to manage the data? | Supplementary material |
| 28 | Participant checking | Did participants provide feedback on the findings? | Supplementary material |
| *Reporting* | | | |
| 29 | Quotations presented | Were participant quotations presented to illustrate the themes/findings? Was each quotation identified? E.g. participant number | Results: Qualitative findings (Table 4);  Supplementary material |
| 30 | Data and findings consistent | Was there consistency between the data presented and the findings? | Results: Qualitative findings;  Supplementary material |
| 31 | Clarity of major themes | Were major themes clearly presented in the findings? | Results: Qualitative findings |
| 32 | Clarity of minor themes | Is there a description of diverse cases or discussion of minor themes? | Results: Qualitative findings;  Supplementary material |

# Part 3: Feasibility of data collection and indicative cost utility analysis

**Aim**

To determine the feasibility of collecting requisite data for a cost utility analysis (CUA) alongside a future RCT.

**Methods**

Using an Intention to Treat approach, an intervention cost was calculated for each arm using surgical staff costs in addition to implant costs calculated using data from a hospital trust’s finance department. Unit costs were obtained from published sources (NHS reference costs, Personal Social Services Research Unit PSSRU data (https://www.pssru.ac.uk/) and the British National Formulary (BNF) and attached to each item of resource use recorded in the survey. The resource use survey required participants to record all NHS resources used during the data collection period including use of any hospital services, primary and community-based services and prescribed medicines. Health-related quality of life (HRQL) scores were assigned to EQ-5D health states reported at baseline and follow-up; QALYs were calculated using these HRQL scores.

In an indicative CUA, an incremental cost-effectiveness ratio (ICER) was calculated by dividing the between-arm difference in costs (adjusted for baseline resource use) by the difference in QALYs (adjusted for baseline HRQL). Incremental net benefit (INB) was calculated by multiplying the between-arm difference in QALYs by the willingness-to-pay threshold used by NICE (£20,000 per QALY) and subtracting the between-arm difference in costs. Pseudo-confidence intervals around the ICER and INB were generated by resampling from the data using bootstrapping with replacement (5000 iterations). The probability of cost-effectiveness at different thresholds was plotted on a cost-effectiveness acceptability curve (CEAC).

**Results**

At baseline, six-week and final follow-up, complete EQ-5D-5L responses, including VAS scores, were obtained from all 36 participants (100%). HRQL scores were assigned to every patient at all time-points.

At the final follow-up, 35 out of the 36 participants provided details of NHS resource use since surgery. Unit costs were identified for each item of resource use and a total resource use cost was calculated.

The mean cost of the intervention in the fully cemented arm was £491.31 (SD: £63.58) and in the hybrid arm was £741.38 (SD: £80.04). Mean per-person resource use cost following surgery was £304.75 (SD: £428.25) in the fully cemented arm and £184.12 (SD: £132.84) in the hybrid arm. The mean number of months between baseline and follow-up was 4.7 (SD: 1.3).

The mean overall per-person improvement in HRQL at the final follow-up was 0.47 (SD: 0.31). The mean improvement in HRQL for the fully cemented arm was 0.40 (SD: 0.33) and in the hybrid arm it was 0.54 (SD: 0.29). Over the six-month time horizon, 0.22 and 0.26 QALYs were generated in the fully cemented and hybrid arms, respectively.

In the base case CUA, the hybrid implant arm generated increased mean costs (+£165) and improved outcomes (+0.034 QALYs) compared to the fully cemented arm. This equates to an ICER of £4,871 (95% centile (bootstrapped) confidence interval: -£47,711 to £64,007) and an INMB of £514 (95% centile (bootstrapped) confidence interval: -£892 to £1,961). Using the threshold of £20,000 per QALY, there was a 76% likelihood of hybrid implants being cost-effective when compared to fully cemented one as shown in the CEAC (figure 1).

Table 1: Indicative cost utility analysis results

|  | Fully Cemented Arm | Hybrid Arm | Difference |
| --- | --- | --- | --- |
| Intervention cost Mean (SD) | £491.31 (£63.58) | £741.38 (£80.04) | £250.07 |
| Per person resource use  Mean (SD) | £304.75 (428.25) | £184.12 (132.84) | -£120.63 |
| Total per person costs | £796.06 (428.25) | £925.49  (132.84) | £129.44 |
| Total per person costs (adjusted for baseline resource use) | - | - | £165.35 |
| Per person QALYs | 0.218 | 0.255 | 0.037 |
| Per person QALYs (adjusted for baseline HRQL) | - | - | 0.034 |
| ICER (pseudo 95% CI) | - | £4871 per QALY (-£47711 to £64007) | - |
| INB (pseudo 95% CI) | - | £514 (£-892 to £1961) | - |

Figure 1: cost-effectiveness acceptability curve

**Discussion**

No missing HRQL scores at any time point indicates the feasibility of collecting EQ-5D data from patients undergoing THR. The EQ-5D is demonstrably sensitive to post-surgery changes in HRQL and can potentially reveal between-arm differences.

The indicative results from the CUA suggesting that, from an NHS perspective, hybrid implants may be preferable to the fully cemented option should be treated with caution given limitations inherent in the exploratory nature of this trial. The relatively high proportion of patients not receiving their allocated treatment (12.5%) should be noted. Moreover, the large uncertainty around ICER estimates, driven by the small sample size, mean that cost-effectiveness estimates in this exploratory analysis are unlikely to provide useful insight about the relative value-for-money of the interventions.

A larger study including a within-trial CUA and economic modelling to account for longer-term impacts could be used to produce a definitive estimate of cost-effectiveness.

# Part 4: Details of qualitative research methods and analysis

***A: Research methods***

**Design:**

Single qualitative, semi-structured interviews were conducted by phone with patients invited to take part in the HipHOP feasibility randomised controlled trial (RCT), consultant orthopaedic surgeons and healthcare professionals involved in running the feasibility trial.

**Patient interviews**

***Patient participants***

Patient participants were eligible for inclusion if they had been approached to take part in the HipHOP RCT and if they had no hearing impairment which would preclude communication by telephone. Participants were purposively sampled to ensure the sample included: patients who accepted and declined the invitation to take part in the RCT; patients from both feasibility trial study sites (sites A and B); patients of various ages (<50 and ≥50 years of age); both female and male patients. A quota-sampling approach to recruitment was taken (Robinson, 2014); we recruited at least two patients with each of the target characteristics. We aimed to recruit between 20 and 30 participants in order to gain a range of perspectives and a rich understanding of participants’ experiences and understanding. Recruitment ceased when the quota sampling criteria were met and data saturation was reached in that no major new topics were arising in interviews. Twenty-seven patients took part in an interview (see Results).

***Patient recruitment***

Patients were first approached about, and invited to take part in, the RCT. Where participants were approached about the RCT during a clinic visit, patients were introduced to the qualitative interview study after completion of RCT discussions, whether or not they were interested in taking part in the RCT, and were provided with the interview study participant information sheet. Where participants were approached about the RCT by mailing study information, with follow-up by phone, the qualitative interview study information sheet was included in the mailed study pack, and interest in the interview study gauged during the telephone call, after discussion of the RCT was complete. Interested participants were asked to give consent for their details to be shared with the academic research team responsible for conducting qualitative study interviews via a ‘details collection form’. This form collected patient contact details and information relevant to the purposive sampling strategy.

Where participants took part in the RCT, the qualitative interview study researcher contacted potential participants by phone at least one week after their surgery. Where participants declined the RCT, participants could be contacted either before surgery, or at least one week after surgery. During this call, the researcher explained the purpose of the qualitative interview study and discussed the participant information sheet with individuals. If the individual was keen to continue to take part in an interview, a date and time for the telephone interview was arranged.

***Patient data collection***

Where participants took part in the RCT, we aimed to conduct qualitative study interviews between 2 and 4 weeks after surgery, although interviews could be completed later (for example, should a patient wish to take part but not feel well enough during this time period). Where participants declined the RCT, interviews could be conducted before surgery (as we did not wish to ask them about experiences of completing RCT questionnaires); if interviews were conducted after surgery, we aimed to interview them 2-4 weeks post-surgery. Informed consent was taken by the researcher by telephone on the interview day, immediately prior to the interview. The researcher reviewed the participant information sheet content with the participant prior to audio-recording consent. The recording was stopped after recording consent, in order to manage and store the consent recording separately to the interview recording. A new audio-recording was started for the study interview.

An interview schedule was used to guide interviews. This was informed by relevant literature and the Theoretical Framework of Acceptability (Sekhon et al 2017). It was reviewed by patient and public involvement contributors and by clinical members of the research team. Topics covered included: patients’ experiences of being approached for the study, their understandings of the study and reasons behind decisions whether or not to participate. Individuals who took part in the RCT were also asked about experiences of taking part in the feasibility trial. Interviews were semi-structured, with open questions designed to ensure that interviews addressed issues in line with the study research questions, but to enable participants to talk freely and discuss issues of importance to them which may not have been considered by the research team.

**Surgeon interviews**

***Surgeon participants***

Surgeon participants were consultant orthopaedic surgeons; a hearing impairment which would preclude communication by telephone was an exclusion criterion. Purposive sampling was conducted to ensure the sample included: surgeons from the two RCT study sites who consented to their patients being randomised for the feasibility trial, RCT study site surgeons who did not consent to their patients being randomised, and surgeons from two additional hospitals (in Northern and Eastern England) which were not participating in the RCT but which could be considered as study sites for a full trial. We aimed to recruit 20-30 surgeon participants to gain a range of perspectives relevant to the research topic, whilst also ensuring a manageable data set to achieve an in-depth and meaningful analysis.

***Surgeon recruitment***

The Principal Investigator or Local Collaborator at each site identified consultant surgeons who would be appropriate for the study. All suitable surgeons at each site were emailed with the study Participant Information Sheet by the HipHOP Chief Investigator (a consultant orthopaedic surgeon). Allowing at least 24 hours to consider the study information, the qualitative interview study researcher followed up each contact to invite questions about the study and to invite surgeons to take part in the study. Two reminders were sent if no response was received. A suitable time for a telephone interview was then arranged with interested individuals.

***Surgeon data collection***

Informed consent was taken using the same procedure as for patient participants. The semi-structured telephone interviews were guided by an interview schedule informed by relevant literature and the Theoretical Framework of Acceptability (Sekhon et al 2017), and reviewed by patient and public involvement contributors and by clinical members of the research team. Topics covered included: beliefs about the surgical options of the HipHOP trial; perceptions and experiences of both research in general and this particular trial; thoughts about appropriateness of randomising patients to this trial; thoughts about issues associated with implementing any guidance that could result from a full trial. Surgeons at RCT study sites were asked about their reasons for allowing, or not allowing, their patients to take part in the feasibility trial. Interviews were audio-recorded.

**Health Care Professional (HCP) participants**

***HCP participants***

These participants were NHS staff who were involved in recruiting participants to the HipHOP RCT and/or in the collection of patient self-reported data for the RCT (e.g. research nurses, registrars). A hearing impairment which would preclude communication by telephone was an exclusion criterion. As this is a small pool of individuals, we aimed to interview all who might be able/willing to take part across the two RCT study sites.

***HCP recruitment***

Appropriate HCPs were identified by the HCP responsible for sharing patient details with the University researcher at each site. Potential participants were emailed by the University researcher with an invitation to take part and a copy of the participant information sheet. This was followed up by email, ensuring HCPs had at least 24 hours to consider the information sheet prior to follow-up contact. Two reminders were sent if no response was received. Questions about the study were answered and dates and times for telephone interviews were arranged as appropriate.

***HCP data collection***

Interviews were conducted as late as was feasible within study timelines to ensure staff had as much experience as possible of recruiting participants and collecting data for the RCT. Informed consent was audio-recorded immediately prior to taking part in the research interview, following the same procedure as for patient interviews. The semi-structured telephone interviews were guided by an interview schedule informed by relevant literature and the Theoretical Framework of Acceptability (Sekhon et al 2017), and reviewed by patient and public involvement contributors and by clinical members of the research team. Topics discussed included: experiences of recruiting patient participants to the HipHOP feasibility trial; thoughts about patient perceptions of the HipHOP study (including thoughts about reasons for patients agreeing or declining participation); experiences of data collection and HCPs’ own thoughts about the HipHOP study. Interviews were audio-recorded.

**Interviewer information**

All interviews were conducted by author AD, a female research assistant employed to conduct this research. Only the research participant was invited to join the interview with AD. AD had been trained in health psychology and research methods to MSc level, with experience of conducting qualitative research in previous research assistant roles. AD was supervised by RP, an experienced academic researcher with a PhD in health psychology. AD had no previous relationship with any of the research participants prior to the study start. On a previous project she had interviewed participants who had undergone cancer-related surgery, but was new to working in the area of hip replacement surgery and had few preconceptions about what the study might find. Research participants were informed that interviews were being conducted, and data managed, by University of Manchester researchers. Participants were informed that members of the research team at other institutions (e.g. clinical research team members) would not be able to access full interview transcripts to reassure participants that members of their health care team (patient participants) or clinical colleagues (HCPs and surgeon participants) would not be able to identify them within the dataset.

**Analysis – all participant groups**

Field notes were made after interviews by the researcher. Interview audio-recordings were transcribed verbatim by a professional transcription company. All transcripts were checked for accuracy against the audio-recordings and identifying details were removed. New identifiers were created (randomised generation of letters) for use in reporting findings.

Our research and analysis approach was consistent with a critical realist perspective: the view that there is a reality which is not dependent on people’s perceptions of it, but that to learn about that reality we need to understand people’s experiences and interpretations of it (Ormston et al 2014). A thematic analysis was conducted, aiming to identify and understand ‘patterns’ within the dataset (Braun & Clarke, 2006). Within the aims of the research, an inductive, data-driven approach was taken: we wished to focus on the participants’ voices, and understand the issues that appeared to be of importance to them. Thus, whilst the Theoretical Framework of Acceptability (Sekhon et al 2017) was used to inform interview schedules, it was not used to structure the analysis. We aimed to understand the experiences and perceptions of all groups of participants in relation to the HipHOP feasibility trial (patients, surgeons and HCPs), and also to understand surgeons’ perspectives regarding implementation of the findings of a full trial. The analysis process was structured using the Framework approach (Ritchie & Spencer, 1994; Spencer et al. 2014). This is a systematic and transparent process which enables other researchers within a team to follow the analysis process and decisions taken by an analyst.

One analyst (AD) familiarised herself with the whole dataset by reading and re-reading transcripts, and noting ideas and thoughts on the transcripts. A second analyst (RP) also read all transcripts and made notes of key ideas and issues for each participant. AD coded a sample of transcripts for each patient group: transcripts were re-read, and codes (labels - brief descriptions of content believed to be of importance) were attached to data. This was carried out with six patient, five surgeon, and two HCP transcripts. These codes were brought together into lists of codes, with codes grouped around similar issues or topics. Remaining transcripts within each group were carefully re-read, seeking ideas which were not covered by this initial list of codes. Any new ideas were coded and added to the lists of codes. These lists were further refined into lists of hierarchically organised lists of categories and sub-categories – working ‘thematic frameworks’. Initially, each participant group’s dataset was examined separately, but it became clear that similarities existed across the datasets, and similar issues were of importance to different groups in some areas of relevance to the study. We therefore decided to bring the data from all participant groups together at this stage in the analysis, to enable a multi-perspective analysis of issues to be taken when relevant. Working thematic frameworks from the three groups were therefore integrated into a single working thematic framework containing hierarchical categories and subcategories of codes (see p.6 below for working thematic framework). These early analysis phases were conducted by AD, with weekly meetings with RP to discuss progress, ideas, and refinement of the working thematic frameworks. RP also commented on and contributed to re-working of the early thematic frameworks into a single working framework for the full dataset. This working thematic framework was then discussed in a meeting with other members of the research team (including clinical members), allowing the analysts to check if the contents of the framework rang true for other members of the research team, and to gain initial insights on potential interpretations of the dataset.

AD used this combined working thematic framework to ‘index’ all transcripts: the framework was systematically applied to the transcripts, noting relevant categories for passages within the data on transcripts using Word. Charting was then carried out by AD and RP: matrices were created in Excel, with column headings mapping onto categories/sub-categories of the working thematic framework, and a row for each participant. Within cells, summaries of relevant data content, with transcript line numbers, were provided. These charts enabled examination of the dataset both across topics for individual participants, and across participants for each topic, with line numbers ensuring ease of movement between charts and the full dataset to ensure that understanding of data summaries occurred in the context of the interview. These charts enabled the mapping and interpretation stage of analyses led by RP: charts were interrogated to gain a fuller and deeper understanding of the dataset, aiding the development of themes and writing of the analysis. Throughout analysis, AD and RP regularly met to discuss the analysis process and to consider the importance and relevance of issues identified. Preliminary findings were shared and discussed with the full research team to gain a wider perspective, informing further understanding of issues, prior to themes being finalised. Writing up of findings was drafted by RP, prior to review and comment from research team colleagues.

Research staff recruiting patients for the RCT recorded given reasons for declining to participate in the RCT; these reasons are presented and considered within the present analysis.

Transcripts were not shared with participants, and participants were not asked to give feedback on study findings in order to minimise burden for participants, especially the value of this practice is under debate (Thomas, 2017)

**References**

Braun, V., & Clarke, V. (2006). Using thematic analysis in psychology. *Qualitative Research in Psychology, 3*, 77-101.

Ormston, R., Spencer, L., Barnard, M., & Snape, D. (2014). The foundations of qualitative research. In J. Ritchie, J. Lewis, C. McNaughton Nicholls, & R. Ormston (Eds.), *Qualitative Research Practice: A Guide for Social Science Students & Researchers* (pp. 1-25). London: SAGE Publications Ltd.

Ritchie, J., & Spencer, L. (1994). Qualitative data analysis for applied policy research. In A. Bryman & R. G. Burgess (Eds.), *Analysing Qualitative Data* (pp. 173-194). London: Routledge.

Robinson, O. C. (2014). Sampling in interview-based qualitative research: A theoretical and practical guide. *Qualitative research in psychology, 11*(1), 25-41. doi:10.1080/1480887.2013.801543

Sekhon, M., Cartwright, M., & Francis, J. (2017). Acceptability of healthcare interventions: An overview of reviews and development of a theoretical framework. *BMC Health Services Research, 17*, 88. doi:10.1186/s12913-017-2031-8

Spencer, L., Ritchie, J., Ormston, R., O'Connor, W., & Barnard, M. (2014). Analysis: principles and processes. In J. Ritchie, J. Lewis, C. McNaughton Nicholls, & R. Ormston (Eds.), *Qualitative Research Practice: A Guide for Social Science Students & Researchers* (pp. 267-293). London: SAGE Publications Ltd.

Thomas, D. R. (2017). Feedback from research participants: Are member checks useful in qualitative research? *Qualitative Research in Psychology, 14*, 23-41. doi:10.1080/14780887.2016.1219435

***B. Working Thematic Framework***

**1. Patient background** (single code for indexing – not currently charted as not central to current research aims)

- Physical activity levels pre-surgery
- Thoughts on biological/physiological factors in regards to hip problem
  - Thoughts regarding age
  - Thoughts regarding weight
- Experiences leading up to surgery
  - Pain before surgery
    - Experience of pain
    - Impact of pain/discomfort
    - Activity limitation
    - Impact on participation
  - Negative impact hip problem on day-to-day activities
  - Financial impact
  - Mental health decline
  - Impact of COVID
  - Social factors
  - Issues around identification/diagnosis of hip problem

**2. Experiences of surgery and recovery** (single code for indexing – not currently charted as not central to current research aims)

- Reasons for surgery
  - Pain
  - Activity limitation
  - Social participation
  - Impacting ability to take part in sports/recreational activities
  - Pain
  - Social influencers
  - Weighing up pros v cons
  - Professional advice
- Concerns around surgery
  - Concerns about anaesthesia / sedation
  - Concerns due to family past experience e.g. blood clot due to hip replacement
  - No concerns
  - Previous experiences
  - COVID related concerns
- Experiences of hospitalisation
  - Experiences with staff
  - COVID impact
- Experiences of recovery
  - Pain post-surgery
  - Regaining activity
  - How long was recovery
  - Guidance around recovery
  - Support during recovery
  - Post-surgical pain experiences
  - Importance of physiotherapy
  - Mood during recovery

**3. Initial approach and introduction to HipHOP**

**3.1 Initial contact**

3.1.1 By whom & how contacted

3.1.2 Views on method of contact

**3.2 Patient discussions with staff**

3.2.1 Verbal information on study

3.2.1.1 HCP strategies to provide information

3.2.2 Reassurance around participation

3.2.3 Views on time/burden of discussions

3.2.4 Misunderstandings about study

3.2.5 Ability to ask questions

3.2.6 Understanding of HipHOP following discussions

3.2.7 Surgeon’s role

3.2.8 Views on explaining trial to patients

**3.3 Experiences around consent**

3.3.1 Novel method of consenting patients

3.3.2 Issues during consenting

**3.4 Views on patients’ study information**

3.4.1 Amount of information provided

3.4.2 Time given to digest information

3.4.3 Feelings on written information

3.4.4 Understanding of study information

3.4.5 Views on information content

**4. Information about hip surgery**

**4.1 Surgeon views on information provided to patients on hip surgery**

**4.2 Patient understanding of & views on the surgical procedure**

4.2.1 Research into procedure

4.2.2 Feelings on understanding of procedure

**5. Decision process around participation in study**

**5.1 Perceptions around benefits to taking part**

5.1.1 Personal benefits

5.1.2 Benefits to others

5.1.3 Benefits to systems

5.1.4 Moral duty to take part

**5.2 Social influence**

5.2.1 Influence of surgeon

5.2.2 Trust in surgeon

5.2.2.1 Appreciated contact from surgeon

5.2.3 Influence of family

**5.3 Previous hip replacement**

**5.4 Concerns regarding participation**

5.4.1 Confidentiality

5.4.2 Research/experimentation

5.4.3 Concerns re study/burden

5.4.4 Felt clinical decision would be more personal

5.4.5 Concerns around surgery

5.4.6 No concerns

**5.5 Other factors in decision making process**

5.5.1 No reason not to

5.5.2 Understanding they can withdraw from study

5.5.3 Understanding of HipHOP

5.5.3.1 Feelings influenced by research

5.5.3.2 Reassuring factors for patients

5.5.4 Context of specialised research hospital

5.5.5 COVID

**5.6 Experiences of declining**

**6. Views on randomization**

**6.1 Fairness of randomization**

6.1.1 Ethics of randomization

6.1.2 Views on surgeon’s role in randomization

**6.2 Understanding of randomization in study**

6.2.1 Views on purpose of randomisation in research design

6.2.2 Understanding of (computerized) randomization

6.2.3 Declining participation due to randomization

6.2.4 Views on being blinded

6.2.5 Feelings towards being randomized

**6.3 HCP thoughts on patients’ views/reactions to idea of randomization**

**7. Equipoise**

**7.1 Patient views on equipoise**

7.1.1 No opinion on which hip might be better

7.1.2 Patient view on whether one is better

7.1.3 Introducing concept of equipoise to patients

7.1.4 Other factors influencing patient views on randomization & equipoise

7.1.4.1 Impact of job role

7.1.5 Patient perception of views of surgeon/HCP

7.1.5.1 Surgeon/HCP did not indicate preference one way or another

7.1.5.2 Surgeon/HCP suggested preference

**7.2 Seeking information on fixation methods**

**7.3 Concerns regarding participation**

7.3.1 Did not like idea of randomization

7.3.2 Previous hip replacement, wanted the same

7.3.3 Wanted surgeon decision based on patient factors (including: patient reassurance if knew that surgeon would withdraw them from study if believed one type would be better)

**7.4 Surgeon /HCP views on equipoise**

7.4.1 Views on whether there is equipoise

7.4.1.1 Views towards randomizing their patients

7.4.1.2 HCP trust in surgeon

7.4.2 Implications of lack of equipoise

7.4.3 Surgeon’s capability in both fixation approaches

7.4.3.1 Having other surgeon conduct surgery if not capable

**8. Experiences of taking part in study**

**8.1 Views on the questionnaires**

8.1.1 Views on time/burden of taking part in HipHOP

8.1.1.1 HCP views on measures

8.1.2 Impact of COVID

8.1.2.1 Understanding of questions

8.1.3 Method/efficiency of data collection

8.1.4 Setting and timing of questionnaires

8.1.5 Support issues

8.1.6 Pleasant experience

8.1.7 Negative experiences

**8.2 Views on follow-up questionnaires**

8.2.1 Concerns around support

8.2.2 No concerns/happy to complete follow ups

8.2.3 Should be long term study

**8.3 Views on burden of research activities**

8.3.1 Travel issues

8.3.2 Hearing (telephone interview, did not impact questionnaire completion)

8.3.3 Feelings around paperwork

8.3.4 Pain

**9. Surgeon views on fixation method**

**9.1 Views on & experiences with fully cemented vs hybrid**

9.1.1 Experiences with the two approaches

9.1.2 Views on resources

**9.2 Factors influencing views/preferences on fully cemented vs hybrid**

9.2.1 Training and experience

9.2.2 Published data

9.2.3 Personal experience/data

9.2.4 Views on longevity of implant

**9.3 Patient factors in deciding method of implantation**

9.3.1 Age/gender of patient

9.3.2 Activity levels of patient

9.3.3 ‘Higher demand’ patients

**10. Views on HipHOP**

**10.1 Views on running trial**

**10.2 Views and experiences of the feasibility study**

10.2.1 Experiences of taking part in HipHOP feasibility

10.2.1.1 COVID impact

10.2.1.2 Experiences discussing trial with patients

10.2.2 Importance of feasibility/pilot before full trial

**10.3 Thoughts on taking part in full trial**

10.3.1 Thoughts on introducing study to patient in full trial

10.3.2 Willingness to take part in full trial

10.3.3 Changing practice

10.3.4 Practical considerations if taking part

**10.4 Views on following study protocol**

10.4.1 Challenges following protocol

10.4.2 Impact on day-to-day working

**11. Concerns regarding HipHOP**

**11.1 Concerns around study design/research methods**

**11.2 Concerns regarding health economics**

**11.3 Inclusion criteria concerns**

**11.4 Thoughts on what HipHOP results will show**

**12. Views on research**

**12.1 Experience in research generally**

**12.2 Views on clinicians taking part in research**

**13. Implementation issues**

**13.1 Surgeon’s beliefs and attitudes**

13.1.1 Difficulties changing strongly held beliefs

13.1.2 Thoughts on strategies to change beliefs

**13.2 Training issues**

13.2.1 Implementation difficult if surgeons only trained/experienced in one approach

13.2.2 Training to facilitate implementation

**13.3 Impact of study findings in changing practice**

13.3.1 Willingness to change practice based on evidence

13.3.2 Strength of findings

**14. HCP background**

**14.1 Experience with patient group**

**14.2 Experience recruiting to trials**

14.2.1 Level of experience recruiting to trials made recruiting easier

14.2.2 Type of trials

**14.3 Level of involvement in HipHOP**

**15. Views on & experiences of recruiting to HipHOP**

**15.1 How staff found recruiting to HipHOP**

15.1.1 Impact of COVID

15.1.2 Admin support

**15.2 HCP experiences with surgeons**

**15.3 HCP information seeking/training**

**16. Views on HipHOP data collection**

**16.1 Views on improving data collection**

**16.2 Issues around follow-ups**

16.2.1 Potential resolutions to issues

**16.3 Views on intra-op data form**

**17. Miscellaneous**

**17.1 HCP Issues around having to discuss different studies**

**17.2 Surgeon views on implications of commercialising hip implants**

**17.3 Geographical differences in preferred approaches**

**17.4 Issues related to participation in qualitative interview study**

# Part 5: Additional qualitative findings details

**Additional sample information**

In describing the sample, caution is taking in reporting characteristics to ensure that anonymity is maintained given the small pools of potential participants across groups.

***Patient sample***

Of the 63 participants approached to take part in the HipHOP RCT, 55 were approached about taking part in a qualitative interview (7 were not approached about an interview because sampling criteria had been filled, and one individual was missed (site oversite)). Of these, 38 (69% of 55) expressed willingness to participate in an interview. Reasons for declining an interview were: no clear reason (**n=12;** including: no reason/no response (8), stating not interested (2), declined after discussion with family (1), consented to the RCT (1)); too busy (work/caring commitments) (**2**); feels too old (**1**); hearing difficulties affecting phone usage **(2)**.

Twenty-eight patients were invited to take part in an interview by the University researcher, in line with the sampling protocol. Interviews were conducted February – August 2021. Twenty-seven patients went on to participate in an interview (one initially agreed to take part but cancelled due to other commitments).

***Surgeon sample***

Forty surgeons were invited to take part across four sites: two were sites participating in the HipHOP feasibility trial; two were not participating in the HipHOP feasibility trial but were potential sites for a future full trial (‘interview-only’ sites). Sixteen surgeons took part in an interview (40% response rate). All study sites were represented in the sample. Interviews were conducted between May and August 2021.

***HCP sample***

All 11 HCPs involved in participant recruitment and/or patient-report data collection for the HipHOP feasibility trial were invited to take part. Four agreed to take part in an interview (36% of 11). Two responded to say they did not have time to take part in an interview; no response was received from the remainder. Both feasibility trial sites were represented in the sample. Interviews were conducted between June and September 2021.

**Additional qualitative analysis findings**

The themes ‘Acceptability of data collection’ and ‘Desire to help’ are briefly summarised in the main text; here, full content for these themes is provided, with supporting quotations.

**Acceptability of data collection**

Most ‘acceptor’ participants seemed to be happy with the processes of taking part in the trial, and seemed to have few concerns about participating:

*It’s all been easy to do, it’s been pleasant, you know, people are nice, they explain things really well […] It’s not an issue really, it’s not, you know, it’s not hard.* (Patient KS, acceptor)

*I just thought, you know what, there’s nothing to lose about doing this*. (Patient SG, acceptor)

The main activity for participants which would take additional time or effort to receiving usual care was completing questionnaires, and these questionnaires were managed such that patients were not required to make additional hospital visits to those required for usual care. The level of involvement required seemed to generally be acceptable to ‘acceptor’ participants, and HCP responses also suggested that questionnaire completion was broadly acceptable:

*it’s not a big deal is it, I have to fill some form and some point in the future, […] It’s just something what’s an occasional thing, so I thought well there’s no problem right, if you were going back to the hospital every week to be put through the third degree like I might have refused it then. But you know, something so infrequent as this it doesn’t matter does it.* (Patient JA, acceptor)

*I think we had one patient who said no because I think [they] – couldn’t see very well or something, just found questionnaires hard, but most people are kind of happy to do questionnaires.* (HCP C)

Some HCP participants reported perceiving that extra hospital visits would have been off-putting; similarly, there were patient participants who indicated they would have been discouraged from participating were additional visits required:

*[…] many people did ask, “do I have to have an extra appointment?” Which of course isn’t the case, so I found they were quite reassured when I said “no, no you don’t need an extra appointment” and explained it was questionnaires and that I could either post them or ring them or see them in clinic.* (HCP F)

*it’s not as if I’m having to trail through to [name of hospital], I didn’t want that, I didn’t want to have to keep trailing through to [name of hospital].* (Patient YE, acceptor)

There was some recognition by both ‘acceptor’ and HCP participants that individuals’ other commitments – in particular, work roles – might impact capacity to complete study questionnaires:

*Well because I’m retired, and it gives me more time to help doesn’t it, you know?* [and later:] *obviously if I was going back to work and I was younger, I wouldn’t be able to do it the same* (Patient VO, acceptor)

If individuals were put off taking part in the HipHOP trial due to the time or effort required to complete questionnaires, they may not have wished to take part in an interview, and so their views might not be represented within our interviews. The table in the article shows that two individuals reported that they declined taking part in the trial arm of HipHOP because they were too busy to do so.

It was mentioned within both HCP and ‘acceptor’ responses that the questionnaires seemed to be lengthy, and shorter questionnaires would have been appreciated.

*Some of the questions – and I ended up laughing and we were laughing about it, but we just got through it. And [the HCP] kept saying I’m really sorry there’s another page, there’s another page.* [and later: ] *I can’t remember what questions there were but, um, some of them seemed quite repetitive.* (Patient YE, acceptor)

However, some felt that the questions asked were relevant and important; there seemed to be recognition of a balance between questionnaire length and the collection of useful information:

*Yeah, it’s – obviously there’s a lot of thought gone into the number of questions and the type of questions. But my experience was – is that, you know, every single one of them was relevant.* (Patient OC, acceptor)

*Would have liked less questions, but then again you don’t get what you need by not having the [questions]. They need to be in there. They’re all relevant*. (HCP Q)

At the time of the qualitative interviews, ‘acceptor’ participants had completed baseline, pre-operative questionnaires, and typically had completed these at hospital, during clinics or when attending for surgery, with trial staff being available to support them. For some, these procedures seemed to work well, particularly for individuals who valued staff support in reading and understanding questions:

*I think they dealt with it incredibly well, you get your own little room, [the HCP] was there, [they] said if you need me just ask me anything. Yeah, it was great, it didn’t take long at all.* (Patient ZR, acceptor)

*[The HCP] made it a lot easier than having me to sit down and read it and maybe miss something.* [and shortly after]: *if I’d have had to read it, I’d have had to take it home and study it for days. But [the HCP] made it very, very clear and I thought that was, well, an absolute plus.* (Patient JU, acceptor),

However, some ‘acceptor’ participants seemed to find juggling the task of completing questionnaires alongside managing other hospital demands challenging and stressful:

*I probably had four or five people in my room [on the morning of surgery] all wanting me to do different bits of paperwork. So I was like, “Can I just have a minute to read one thing at a time?” […] you just need a bit of time and I didn’t get it.* (Patient NL, acceptor)

The perceived long length of questionnaires seemed to compound the challenges of completing them whilst at the hospital for other purposes:

*Well, it’s just that they’re so long. […] I was asked after I’d seen the surgeon, and I’d been waiting there for x-rays and things. In the end I was at the hospital about three hours […] And doing it when you’ve just done all that assessment, it was a bit long winded, if you know what I mean. […] it was just a bit long at that stage*. (Patient UG, acceptor)

This participant seemed to feel that the questionnaires contributed to an over-long hospital visit, and there is a sense in the above quote that, after completing assessments, they may have been tired by the time they started on the study questionnaires.

Patient interviews included discussion of how participants felt about being asked to complete follow-up questionnaires. Many individuals seemed comfortable with this prospect:

*I’m sure I’ll be fine with that as well.* (Patient KS, acceptor)

*[…] the next one, which I’ll have at home I assume, and then I can sit down and do it in my own time. It’ll probably seem a lot easier because I’m doing it on my own* (Patient UG, acceptor)

As noted by Patient UG, having the follow-up questionnaires at home seemed to have the potential to remove pressures of completing questionnaires alongside activities demanded during hospital visits. One HCP similarly noted challenges of completing lengthy questionnaires, and felt that the questionnaire length might be better suited to follow-up questionnaires, where patients would typically be at home:

*I think that the amount of questions is fine for a post-operatively, because you’re posting them out […] And they can think, oh, okay, I’ll do that after dinner tonight or tomorrow morning or on the weekend.* (HCP W)

Nevertheless, some patients were keen that they would still be able to access support when at home:

*If I had any difficulties, it would be can you ask somebody, you know, filling it in if I don’t understand it? Would I be able to ask somebody?* (Patient FE, acceptor)

‘Decliner’ interview participants generally indicated that they would have been happy to complete questionnaires, that they would have been easy enough, or even enjoyable. It seems that other aspects of the study – issues around randomisation to implant type – were more of an issue for them:

*It’s not an inconvenience filling a questionnaire in, none whatsoever really. I’m quite happy to do that sort of thing. It’s just that I didn’t really want to have a surgical procedure as part of that, yeah.* (Patient BY, decliner)

*If I’d in principle been happy to go random and not know what the detail was, then if I had got past that hurdle, I would have had no problem filling in questionnaires*. (Patient OT, decliner)

*Well, I remember that they did ask me about those questions, about you know, questionnaires. And I was more than happy to do all that, yeah. In fact, I would have enjoyed being part of that study.* (Patient AS, decliner)

Individuals who declined taking part in interviews – as well as the trial arm – may have found the prospect of participating in research unappealing generally, or have had additional concerns. Some HCP participants perceived that patients could feel overwhelmed or stressed on the run-up to surgery, such that they were reluctant to have anything additional – such as research participation - to manage:

*For people that declined, that I managed to have a chat to, it was mostly just the – oh, it’s just – like they were worried about their hip replacement and it’s just a bit overwhelming to add an extra layer to it, that was the main consensus*. (HCP W)

There was also a perception that individuals who were older or more frail might be more likely to decline participation:

*I guess the one person that declined was probably more frail than the others [who did take part]. […] generally does tend to be patients that are maybe older or frailer, or got a few more comorbidities that just don’t want the extra hassle*. (HCP C)

One individual who declined participation in the trial arm of HipHOP gave the reason of feeling too old to trial staff (see Table 3, main text).

**Desire to Help**

For many patient participants, the desire to help improve treatment for people in the future seemed to be important and influential in their decision to take part in the trial:

*I’m thinking, well, if I can help to make things easier in the future for somebody else, then do it, just do it.* (Patient KS, acceptor)

Some seemed to feel that they were receiving the procedure anyway, so if as part of that process they could help other patients as well, then that seemed perceived to provide additional value:

*if I’m having it done I might as well make the best of it, and you know, make it better for others as well*. (Patient SG, acceptor)

Some individuals seemed to perceive scientific research to be important, and appeared to be happy to be involved in the scientific process:

*medical testing is what you have to do to prove that things work or don't work. So I think it's nice to be involved in things like that.* (Patient AS , decliner)

*I think it’s, you know, inevitably with this kind of surgery that the more data there is, the better […] So any research that gives patients peace of mind or gives surgeons and consultants and staff, greater insight into the best techniques, then any research which supports that definitely has got to be the right thing to do, hasn’t it?* (Patient OT, decliner)

It is notable that the above pro-research quotes are from individuals who declined taking part in the trial. It seemed that an enthusiasm for participating in research in general could lead to some conflict for ‘decliners’. Some seemed to be balancing a desire to support research in general against concerns about taking part in this specific RCT. In one case, having the opportunity to take part in an interview without taking part in the trial seemed to almost be a relief, allowing them to support the overall research without compromising their reluctance to participate in the RCT:

*and I kind of raised my concerns […] [the HCP] said, "There are two parts to it, so you can actually go on part two [the qualitative interview] to see why." I said, "That'd be great, so I'll do that." 'Cos I want – you kind of do want to take part, because obviously you want to, you know, help where you can. [and later:] [the HCP] could see the kind of unsureness in my voice, and […] let me feel at ease to be quite happy to say, "No, it's all right, but I will take part in something else." So, I kind of felt I was still doing something.* (Patient RE, decliner)

Some patients considered the value of their participation to the NHS, and expressed a desire to support the NHS:

*it’s not going to cost you anything, it’s just, you know, just do it, it’s helping the NHS out isn’t it?* (Patient JA, acceptor)

For some patient participants, there appeared to be a sense of obligation around taking part in the research. Individuals seemed to value receiving their hip replacement surgery from the NHS and their care teams, and seemed glad to be able to reciprocate:

*it’s a two way street, the NHS are helping me get back on my feet and hopefully improving my quality of life, so all I want to do is actually give back* (Patient ZR, acceptor)

*it seemed like it was the least I could do for having the opportunity to have the operation, given how many people are on the waiting list at the moment.* (Patient FB, acceptor)

*[name of surgeon] was wonderful, and I’d like to help [surgeon] best way I can* (Patient VO, acceptor)

This sense of reciprocity was also observed by HCP participants:

*The priority was to help. That’s something that I’ve heard from most patients. Yes, anything to help, why not […] a lot of people genuinely do want to help because they appreciate the treatment that they do have.* (HCP F)

*Quite a lot of people said, oh, it’s nice to help. I like to do my bit. […] yeah, they definitely liked being able to give back to the service that was helping them.* (HCP W)

Some seemed to go as far as to consider participating a moral obligation, both in recognition of how the NHS supported them, and out of obligation to society more widely:

*I felt it a moral duty to take part in the study, given that I had the opportunity to have the operation under the National Health Service, and to improve my quality of life, it was a little to give back, if you like, for the service that had been provided to me*. (Patient FB, acceptor)

*I feel like I’m part of like a – want to be part of a community and to be part of the community you have to give.* (Patient ZR, acceptor)

There was also a belief that the surgery patients had received was the result of people taking part in research previously, such that there was a sense that they were now able to contribute to the scientific research process themselves, to enhance treatment for future patients:

*people have done something before that probably has helped me, so I just feel if I can give something back maybe, want somebody in the future, then I just think it’s a good thing to do.* (Patient KS, acceptor)
